# Killer cell immunoglobulin-like receptor 2DL4 is expressed in and suppresses the cell growth of Langerhans cell histiocytosis

**DOI:** 10.18632/oncotarget.16936

**Published:** 2017-04-07

**Authors:** Yusuke Takei, Chiyuki Ueshima, Tatsuki R. Kataoka, Masahiro Hirata, Akihiko Sugimoto, Mariyo Rokutan-Kurata, Koki Moriyoshi, Kazuo Ono, Ichiro Murakami, Sanju Iwamoto, Hironori Haga

**Affiliations:** ^1^ Department of Diagnostic Pathology, Kyoto University Hospital, Kyoto, Japan; ^2^ Department of Diagnostic Pathology, Kyoto Medical Center, Kyoto, Japan; ^3^ Department of Pathology, Japan Red Cross Society Wakayama Medical Center, Wakayama, Japan; ^4^ Department of Pathology, School of Medicine, Kochi University Faculty of Medicine, Nankoku, Japan; ^5^ Department of Pharmacology, Toxicology & Therapeutics, Division of Physiology & Pathology, Showa University School of Pharmacy, Tokyo, Japan

**Keywords:** KIR2DL4, inhibitory receptor, Langerhans cell histiocytosis, ERK, SHP-2, Pathology Section

## Abstract

Killer cell immunoglobulin-like receptor (KIR) 2DL4 (CD158d) is a receptor for human leukocyte antigen-G. The function of KIR2DL4 has been reported in human natural killer cell lymphoma and mastocytosis, but not in Langerhans cell histiocytosis (LCH). Herein, we examined the expression and function of KIR2DL4 in LCHs. In pathological specimens, 27 of 36 LCH cases (75.0%) were immunohistochemically positive for KIR2DL4. Its expression was independent of age, gender, location, multi- or single-system, and the status of BRAFV600E immunostaining. We also confirmed the expression of KIR2DL4 mRNA and protein in the human LCH-like cell lines ELD-1 and PRU-1. KIR2DL4 protein was distributed in the membrane and cytoplasm of ELD-1 cells, but only in the cytoplasm of PRU-1 cells. An agonistic antibody against KIR2DL4 reduced phosphorylation of extracellular signal-regulated kinases (ERKs) and suppressed the cell growth of ELD-1 cells in a Src homology region 2 domain-containing phosphatase-2 dependent manner, but it had no effect in PRU-1 cells. These results suggest that KIR2DL4-mediated ERK suppression is a possible therapeutic target for LCH cells.

## INTRODUCTION

Killer cell Ig-like receptors (KIRs) recognize major histocompatibility complex class I molecules and regulate human natural killer (NK) cell function both positively and negatively [1, 2]. KIRs are categorized into two groups: KIRs with long cytoplasmic domain (L) containing immunoreceptor tyrosine-based inhibitory motifs (ITIMs) or KIRs with short cytoplasmic domain (S) deficient in ITIMs. KIR2DL4 (CD158d) has a long cytoplasmic domain and contains ITIMs, which can transduce inhibitory signals to NK cells [3]. The inhibitory signals by KIR2DL4 are mediated by Src homology region 2 domain-containing phosphatase (SHP)-1 and SHP-2 in NK cells [3]. Despite the existence of ITIMs, some reports indicate that KIR2DL4 enhances NK activity and induces interferon-γ secretion [4–7]. The ligand of KIR2DL4 is identified as human leukocyte antigen (HLA)-G, whose expression is observed in fetal tissues during pregnancy [1, 2]. KIR2DL4 seems to be involved in the maintenance of pregnancy [8, 9]. Recently, we observed the expression of KIR2DL4 in human mast cells [10]. KIR2DL4 in human tissue mast cells enhances metastasis and invasion of HLA-G-positive cancer cells [10]. Additionally, NK cell lymphoma and neoplastic mast cells (mastocytosis) express KIR2DL4 immunohistochemically [10, 11]. To the best of our knowledge, the expression or role of KIR2DL4 has not been clarified in human non-NK or non-mast cell lineage malignancies.

Langerhans cell histiocytosis (LCH) is considered a neoplasia of Langerhans cells or a histiocytic tumor expressing S-100, CD1a and CD207 [12]. Some reports suggest that LCH cells are more closely related to myeloid dendritic cell precursors than to Langerhans cells [13]. Gene mutations in LCH have been observed in the RAS - RAF - MAP2K (mitogen-activated protein kinase kinase) - MAPK (mitogen-activated protein kinase) signal pathway, including BRAFV600E or MAP2K1 mutations [14–16]. These mutations result in the activation of extracellular signal-regulated kinases (ERKs) and seem to be associated with the common pathogenesis of LCH [16]. In this report, we analyze the expression and function of KIR2DL4 in human LCH cells.

## RESULTS

### Expression of KIR2DL4 in LCH

**Figure 1:**
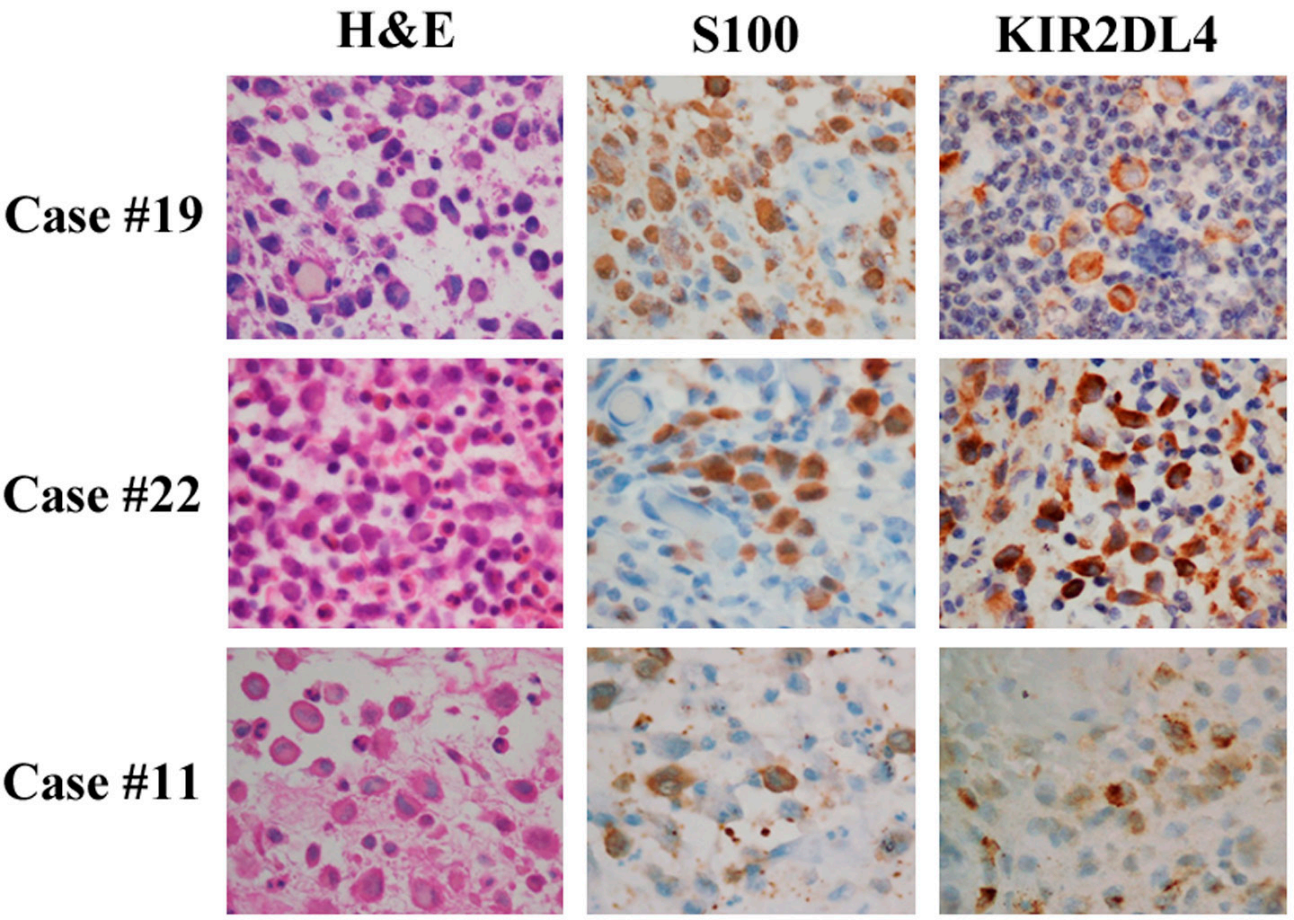
KIR2DL4 protein is expressed in pathological samples of Langerhans cell histiocytosis Three representative cases are presented here. Cases #19 and #22 exhibited positive staining for the cytoplasm and membrane of tumor cells. Case #11 exhibited positive staining for the cytoplasm but negative for the membrane of tumor cells. Immunohistochemistry (x400).

The tumor cells in 27 of 36 LCH cases (75.0%) were immunohistochemically positive for KIR2DL4 (Table 1). Of the positive cases, 24 (88.9%) exhibited both membranous and cytoplasmic staining and the other 3 (11.1%) exhibited cytoplasmic staining only (Figure 1). No clinical parameter (age, gender, location, multi- or single-organ involvement, or BRAFV600E immunostaining positivity) differed between the KIR2DL4-positive and -negative cases.

**Table 1:** Clinical parameters and KIR2DL4 expression status in the LCH cases examined

Cases	Age	Gender	KIR2DL4 expression	BRAFV600E staining	Location/ number of lesion(s)
**#1**	1	f	-	-	Skull/ solitary
**#2**	9	m	-	-	Humerus/ solitary
**#3**	6	m	-	-	Skin (back)/ multiple
**#4**	15	m	-	-	Ilium/ solitary
**#5**	22	m	-	+	Femur/ solitary
**#6**	4	m	-	-	Tibia/ multiple
**#7**	6	f	-	-	Uncertain
**#8**	38	f	-	-	Dura/ solitary
**#9**	62	m	-	-	Maxilla/ multiple
**#10**	0	m	+/-	-	Skin (trunk)/ multiple
**#11**	25	m	+/-	-	Vertebra/ solitary
**#12**	26	m	+/-	+	Vertebra/ multiple
**#13**	4	f	+/+	-	Clavicle/ solitary
**#14**	4	f	+/+	-	Humerus/ solitary
**#15**	23	m	+/+	-	Vertebra/ solitary
**#16**	16	m	+/+	-	Rib/ solitary
**#17**	10	f	+/+	-	Femur/ solitary
**#18**	10	m	+/+	-	Femur/ solitary
**#19**	25	f	+/+	+	Ischium/ solitary
**#20**	42	m	+/+	-	Mandible/ multiple
**#21**	42	m	+/+	+	Mandible/ solitary
**#22**	11	f	+/+	+	Skull/ solitary
**#23**	10	m	+/+	+	Fibia/ solitary
**#24**	10	m	+/+	-	Scapula/ solitary
**#25**	38	f	+/+	+	Lung/ solitary
**#26**	5	f	+/+	-	Femur/ solitary
**#27**	4	f	+/+	-	Femur/ multiple
**#28**	35	m	+/+	-	Lung/ solitary
**#29**	27	m	+/+	-	Skull/ solitary
**#30**	3	f	+/+	-	Femur/ solitary
**#31**	0	m	+/+	-	Skin (shoulder)/ multiple
**#32**	43	m	+/+	-	Lung/ solitary
**#33**	6	f	+/+	-	Skull/ multiple
**#34**	21	f	+/+	-	Lung/ multiple
**#35**	0	m	+/+	+	Skin(back)/ multiple
**#36**	34	m	+/+	-	Mandible/ solitary

The LCH cells of all cases were confirmed to express S-100 and CD1a.

In terms of KIR2DL4 expression “+/-” indicates cytoplasmic positivity but membrane negativity, and “+/+” indicates cytoplasmic and membrane positivity.

### Expression of KIR2DL4 in the LCH-like cell lines ELD-1 and PRU-1

**Figure 2:**
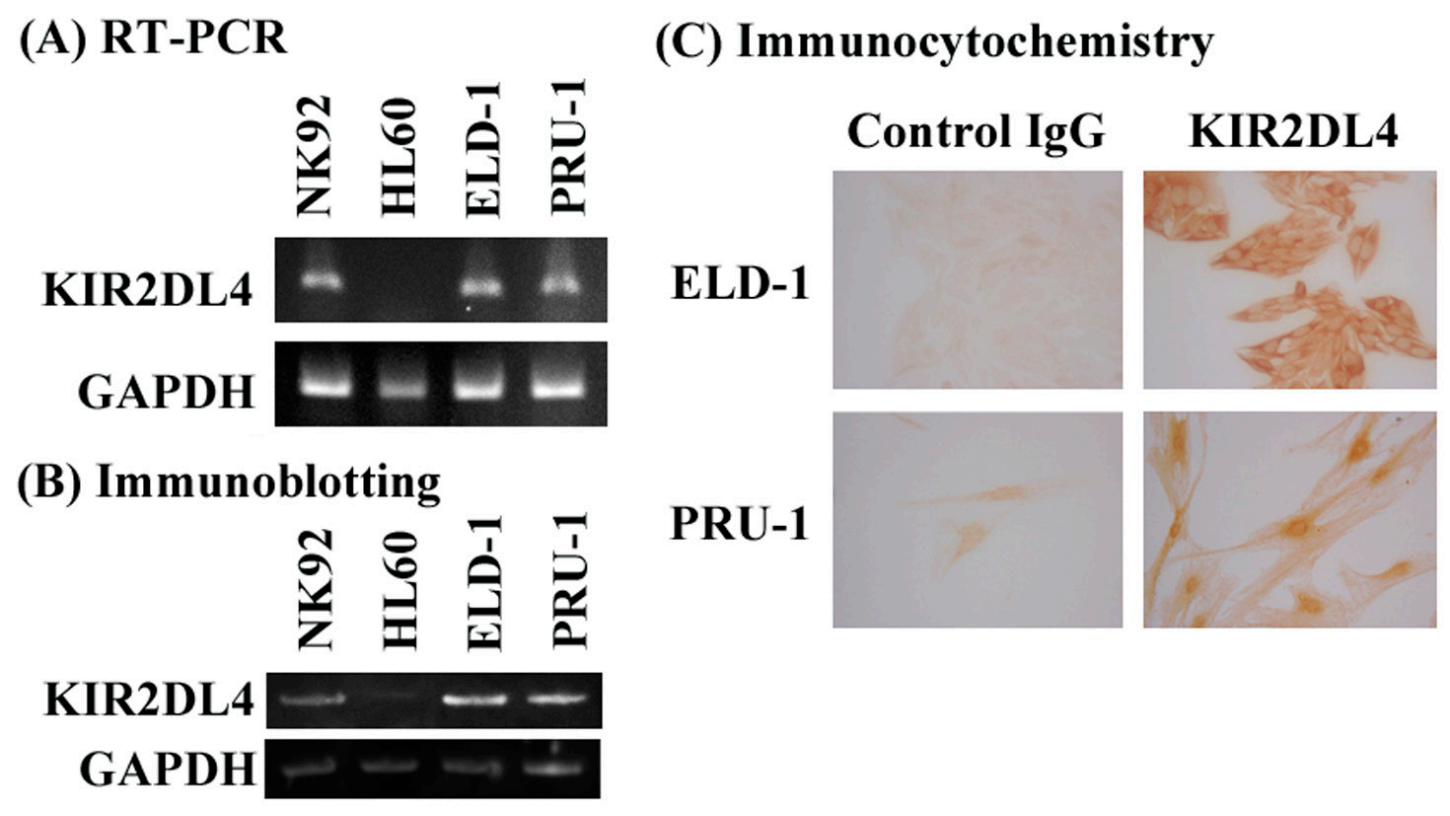
ELD-1 and PRU-1 cells express KIR2DL4 mRNA and protein **A**. RT-PCR, **B**. Western blotting, and **C**. immunocytochemistry were performed as described in the Materials and Methods section. NK92 cells were used as a positive control and HL60 cells were used as a negative control in (A) and (B).

Next, we evaluated KIR2DL4 expression in the LCH-like cell lines ELD-1 and PRU-1 [17, 18]. KIR2DL4 mRNA and protein expression was detected in the both cell lines (Figures 2A & 2B). Immunocytochemical analysis revealed KIR2DL4 protein expression in both the membrane and cytoplasm of ELD-1 cells, but only in the cytoplasm of PRU-1 cells (Figure 2C).

### The effects of an anti-KIR2DL4 agonistic antibody on the cell growth of LCH-like cell lines

**Figure 3:**
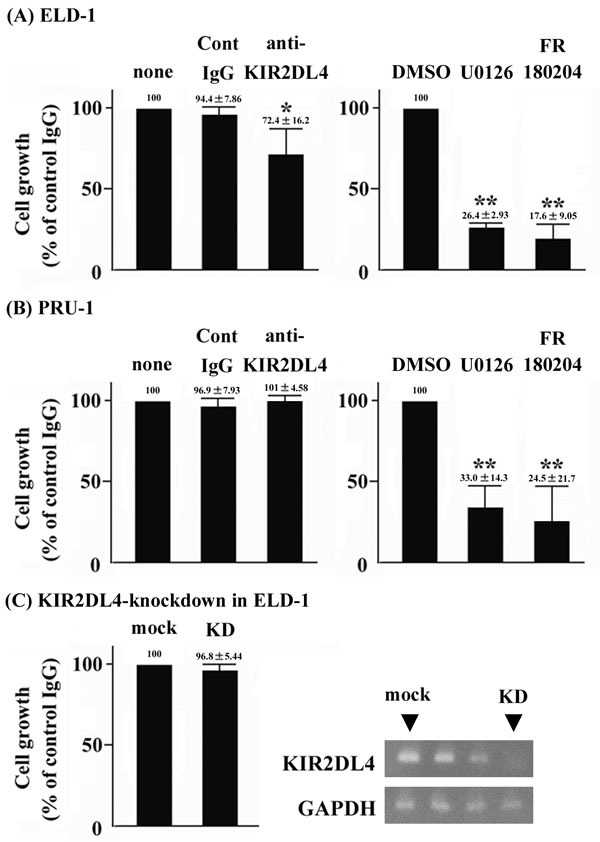
Anti-KIR2DL4 agonistic antibody negatively regulates the cell growth of ELD-1, but not PRU-1 cells Furthermore, KIR2DL4 knockdown in ELD-1 cells does not. ELD-1 and PRU-1 cells were incubated for 24 h with control IgG or anti-KIR2DL4 agonistic antibody (10 μg/ml) (n=3, respectively). ELD-1 and PRU-1 cells were incubated for 24 h with DMSO, U0126 (10 μM) or FR180204 (10 μM) (n=3, respectively). Mock or KIR2DL4-knockdown ELD-1 cells were incubated for 24 h without antibody (n=3, respectively). Growth was assessed as described in Materials and Methods. Relative values are indicated the growth levels of cells with no treatment, those treated with DMSO, or mock cells were set to 100. *p < 0.05 compared with no treatment. **p < 0.05 compared with DMSO treatment.

We used an anti-KIR2DL4 agonistic antibody to explore the roles of KIR2DL4 on the cell growth of LCH-like cell lines. The antibody reduced the cell growth of ELD-1 cells (Figure 3A), but not PRU-1 cells (Figure 3B). Gene mutations in LCH are expected to trigger activation of ERKs [14–16]. Thus, we treated the ELD-1 and PRU-1 cells with the MAP2K1 inhibitor U0126 or the ERK inhibitor FR180204. Both inhibitors dramatically suppressed the cell growth of both cell lines (Figures 3A & 3B). Next, we established KIR2DL4-knockdown ELD-1 cells. The cell growth of KIR2DL4-knockdown ELD-1 was comparable with that of mock ELD-1 in the absence of the anti-KIR2DL4 agonistic antibody (Figure 3C).

### The effects of anti-KIR2DL4 agonistic antibody on signal transduction of ELD-1

**Figure 4:**
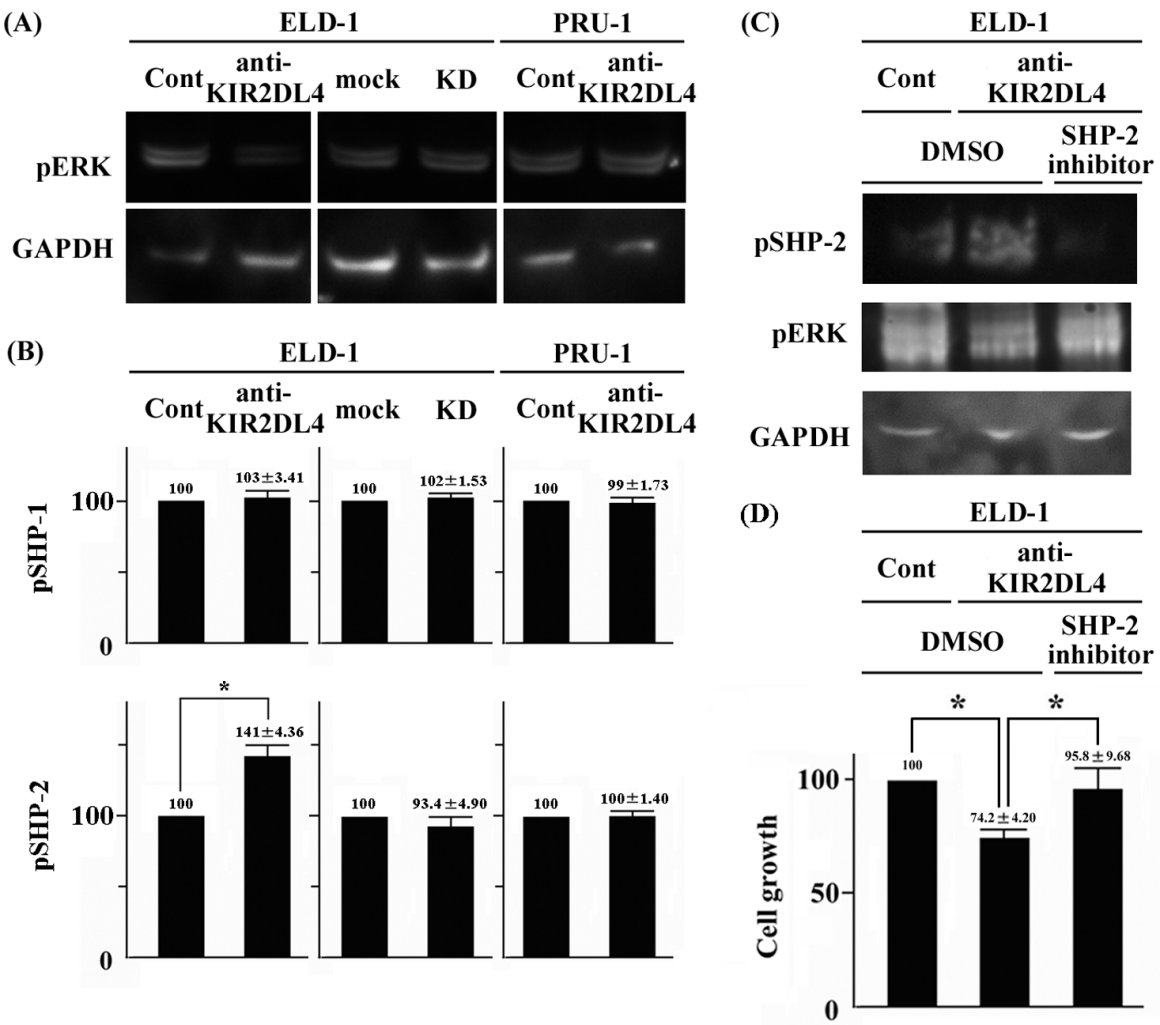
An anti-KIR2DL4 agonistic antibody reduced ERK phosphorylation and cell growth by activating SHP-2 in ELD-1 cells **A**. An anti-KIR2DL4 agonistic antibody reduced phospho-ERK levels in ELD-1 cells. However, the antibody did not reduce phospho-ERK levels in PRU-1 cells, as was also true of KIR2DL4 knockdown in ELD-1 cells. Data are representative of three individual experiments. **B**. An anti-KIR2DL4 agonistic antibody increased phospho-SHP-2 levels in ELD-1 cells. However, the antibody did not decrease phospho-SHP-2 levels in PRU-1 cells, as was also true of KIR2DL4 knockdown in ELD-1 cells. Relative values are based on control IgG or mock values of 100. *p < 0.05 compared with control IgG. **C**. A specific inhibitor of SHP-2, PHPS1, rescued the anti-KIR2DL4 antibody-induced decrease in ERK phosphorylation in ELD-1 cells. Data are representative of those of three individual experiments. **D**. PHPS1 rescued the anti-KIR2DL4 antibody-induced growth reduction in ELD-1 cells. The values are relative to those of the control IgG and DMSO samples (100). *p < 0.05 compared with the anti-KIR2DL4 agonistic antibody and DMSO values.

We evaluated the effects of the anti-KIR2DL4 agonistic antibody on signal transduction of ELD-1. As shown in Figures 3A and 3B, ERK activity was a principal contributor to the growth of LCH-like cell lines. First, we examined the effect of the anti-KIR2DL4 agonistic antibody on the phosphorylation status of ERKs in LCH-like cell lines. The anti-KIR2DL4 agonistic antibody reduced ERK phosphorylation in ELD-1, but not PRU-1 (Figure 4A). The phosphorylation status of the ERKs in KIR2DL4-knockdown ELD-1 cells was comparable to that of mock ELD-1 cells in the absence of the anti-KIR2DL4 agonistic antibody (Figure 4A). The anti-KIR2DL4 agonistic antibody did not affect the phosphorylation status of STAT3, AKT, or Src family kinases (data not shown). The status of these signal molecules in KIR2DL4-knockdown ELD-1 cells was comparable to that of mock ELD-1 cells in the absence of the anti-KIR2DL4 agonistic antibody (data not shown).

SHP-1 and SHP-2 were activated in human NK cells, and only SHP-2 was activated in human mast cells, in the presence of the anti-KIR2DL4 agonistic antibody [3, 10]. We performed ELISA to explore the effect of the anti-KIR2DL4 agonistic antibody on the phosphorylation status of SHP-1 and SHP-2 in LCH-like cell lines. SHP-2 was phosphorylated in ELD-1 and PRU-1 cells even in the absence of any stimulation. The anti-KIR2DL4 agonistic antibody increased the level of phospho-SHP-2 in ELD-1 cells, but not in PRU-1 cells (Figure 4B). The anti-KIR2DL4 agonistic antibody did not affect the phosphorylation status of SHP-1 in either ELD-1 or PRU-1 cells (Figure 4B). The phosphorylation statuses of both SHP-1 and SHP-2 in KIR2DL4-knockdown ELD-1 cells were comparable to those in mock ELD-1 cells in the absence of the anti-KIR2DL4 agonistic antibody (Figure 4B).

SHP-2 activation decreased the anti-KIR2DL4 agonistic antibody-induced ERK activation in human mast cells [10]. Next, we evaluated the association between SHP-2 and ERK phosphorylation statues in ELD-1 cells using a specific inhibitor of SHP-2, PHPS1. This inhibitor rescued the anti-KIR2DL4 antibody-induced decrease in phospho-ERK level in, and the cell growth of ELD-1 (Figure 4C & 4D). We next examined the phosphorylation status of SHP-2 in the clinical LCH samples immunohistochemically. We found no difference in SHP-2 phosphorylation status between KIR2DL4-positive and -negative samples; the cytoplasm was positive for phospho-SHP-2 in all tumor cells (36 of 36 cases [100%], Supplementary Figure 1).

## DISCUSSION

LCH cells express S-100, CD1a, and CD207 [12]. We found that most of the LCH cases were also positive for KIR2DL4, suggesting that KIR2DL4 may serve as an additional marker for LCH diagnosis.

In a few LCH cases, KIR2DL4 protein expression was limited to the cytoplasm. Similarly, the PRU-1 cell line exhibited KIR2DL4 immunoreactivity in the cytoplasm only. The anti-KIR2DL4 agonistic antibody did not affect the growth of PRU-1 cells, perhaps because of the lack of KIR2DL4 protein expression in the membrane, in contrast to ELD-1 cells. We previously detected KIR2DL4 protein in the cytoplasm, but not in the membrane, of the human mastocytosis cell line HMC1.2 and HMC1.2 cell growth was not affected by addition of the anti-KIR2DL4 agonistic antibody [10]. Further studies are required to explore the differences in LCH cases in whom KIR2DL4 protein expression is limited, thus the absent from the membrane.

We found that SHP-2 was consistently phosphorylated in LCH-like cell lines and in all evaluated clinical LCH cases; this was also true of ERKs. Further work is needed to define the mechanism by which SHP-2 is continuously phosphorylated in LCH cells. Mutations in such cells would be expected to trigger continuous ERK phosphorylation [14–16]. Our work in ELD-1 cells suggested that KIR2DL4 influences SHP-2 and ERK statuses, and that cell growth was enhanced only when the ligand (HLA-G) was present. We found no HLA-G-expressing cells in LCH specimens and ELD-1 cells were negative for HLA-G-negative (data not shown). This would explain why no difference in any clinical parameter or phospho-SHP-2 status was evident between KIR2DL4-positive and -negative cases. Together, the data suggest that ERK-induced growth activity is not affected by SHP-2 phosphatase activity with no further stimulation, and that the phospho-SHP-2 level increases only when KIR2DL4 is stimulated by ligand, and this phosphatase activity then reduces the ERK-dependent cell growth of LCH cells.

Thus, the anti-KIR2DL4 agonistic antibody suppressed the growth of LCH cell line ELD-1. This antibody may be a useful therapeutic tool for LCH, as well as other antibodies targeting inhibitory receptors [10, 19–21].

## MATERIALS AND METHODS

### Clinical specimens

Histological specimens diagnosed as LCHs were obtained from Kyoto University Hospital (Sakyo-ku, Kyoto, Japan), Kyoto Medical Center (Fushimi-ku, Kyoto, Japan), and Japan Red Cross Society Wakayama Medical Center (Wakayama, Japan). Patients attending Kyoto University Hospital signed the “Kyoto University Hospital Informed Consent Form for the Non-therapeutic Use of Histopathological Materials”, and the signed forms have been uploaded into all electronic health records. We also obtained written permission from patients attending Kyoto Medical Center or Japan Red Cross Society Wakayama Medical Center. Table 1 summarizes the clinical characteristics of patients.

### Cell lines

We analyzed two cell lines showing common phenotypes with Langerhans cells: ELD-1 and PRU-1 cells [17, 18]. NK-92 was used as a positive control for KIR2DL4 expression and HL60 was used as a negative control [10]. Both cell lines were purchased from the American Type Culture Collection (ATCC, Manassas, VA). NK-92 cells were grown in RPMI1640 medium supplemented with 10% FBS containing recombinant human IL-2. HL60 cells were grown in RPMI1640 medium supplemented with 10% FBS.

### Antibodies and reagents

The anti-KIR2DL4 agonistic antibody (mouse monoclonal IgG, Clone; 181703) was purchased from R&D Systems (Minneapolis, MN). Another anti-KIR2DL4 antibody (rabbit IgG, polyclonal, ab154386) and an anti-phospho-SHP-2 antibody (Y542, rabbit polyclonal IgG), used for immunostaining and Western blotting were obtained from Abcam (Cambridge, MA). An anti-phospho-ERK antibody and an anti-GAPDH antibody were obtained from Cell Signaling Technology (Beverly, MA). The KIR2DL4-targeted shRNA lentiviral particles and the control particles were purchased from Santa Cruz Biotechnology (San Diego, CA). Infection of these viral particles and the following selection were performed according to the manufacturer's instructions. The specific MAP2K1 inhibitor U0126, the specific ERK inhibitor FR180204, and the specific SHP-2 inhibitor PHPS1 were purchased from Santa Cruz Biotechnology [22–24]. We used all inhibitors at concentrations of 10 µM.

### Immunohistochemistry

After deparaffinization with xylene, tissue sections were rehydrated and pre-treated with 0.3% hydrogen peroxide for 5 min. After steam heating for 40 min, the anti-KIR2DL4 antibody (ab154386) or anti-phopsho-SHP-2 antibody was added as primary antibody overnight at 4°C. Staining was performed using the ENVISION kit (HRP, DAKO Cytomation, Glostrup, Denmark) as per the manufacturer's instructions. S100 staining was performed using a Ventana Benchmark Ultra autoimmunostainer (Roche Diagnostics, Mannheim, Germany) according to the manufacturer's protocols. Sections were counterstained with Mayer's hematoxylin solution. Stained sections were imaged using a BX63 microscope fitted with a camera (Olympus, Tokyo, Japan).

### RT-PCR

Cell samples (each 5 × 10^6^ cells) were processed with TRIzol (Invitrogen Life Technologies) overnight at -20°C. Total mRNAs were obtained using RNeasy columns according to the manufacturer's instructions (Invitrogen Life Technologies). A total of 500 ng of each mRNA was reverse-transcribed (SuperScript III One-Step RT-PCR System; Invitrogen Life Technologies). The RT products were used for PCR using primers 5’-CTGTCCCTGAGCTCTACAA-3’ and 5’-CACTGAGTACCTAATCACAG -3’) with the following conditions: 35 cycles of 30 sec at 94°C (denature), 1 min at 60°C (anneal), and 1 min at 72°C (extend), with a final extension for 10 min at 72°C [25].

### Immunoblotting

Cell lysates were prepared and the proteins were separated by electrophoresis. Gels were probed for immunoreactive proteins as described previously [19].

### Immunocytochemistry

ELD-1 or PRU-1 cells were re-cultured in the 8-well chamber glass slides, and fixed in 4% (v/v) paraformaldehyde. The anti-KIR2DL4 antibody (ab154386) or isotype control antibody was added and incubation was continued for 2 h at room temperature. Staining was performed using the ENVISION kit (HRP). Stained cells were imaged using the BX63 microscope fitted with a camera (Olympus).

### Cell growth assay

Cell growth was evaluated using a Cell Counting Kit-8 (CCK-8; Dojindo, Kumamoto, Japan). ELD-1 and PRU-1 cells were cultured for 22 hours at 1-3 × 10^4^ cells/100 µl of RPMI1640 containing 10% FCS with control IgG or anti-KIR2DL4 agonistic antibody (10 µg/ml, each). KIR2DL4-knockdown or mock ELD-1 cells were also cultured for 22 hours at 1-3 × 10^4^ cells/100 µl of RPMI1640 containing 10% FCS. We added 10 µl of CCK-8 solution for the last 2 hours and estimated the absorbance at 450 nm, according to the manufacturer's instructions.

### Assessment of phospho-SHP levels

We measured the amounts of phospho-SHP-1 and phospho-SHP-2 levels using the SHP-1, phospho Tyr536 Colorimetric Cell-Based ELISA Kit (Merck Millipore, Billercia, MA) and the Human / Mouse / Rat Phospho-SHP-2 (Y542) DuoSet (R&D Systems). ELD-1 and PRU-1 cells were cultured for 1 h at 1-3 × 10^4^ cells/100 µl of RPMI1640 containing 10% FCS in the presence of control IgG or anti-KIR2DL4 agonistic antibody (10 µg/ml, each). KIR2DL4-knockdown or mock ELD-1 cells were cultured for 1 h at 1-3 × 10^4^ cells/100 µl of RPMI1640 containing 10% FCS. After centrifugation, cell pellets were processed and subjected to ELISA according to the manufactures’ protocols.

### Statistical analysis

Data were expressed as means ± SE. Differences between groups were examined for statistical significance using Student's *t*-test (Excel: Microsoft, Redmond, WA, USA). A *p* value less than 0.05 indicated statistical significance.

## SUPPLEMENTARY MATERIALS FIGURE



## Abbreviations

ERK: Extracellular signal-regulated kinases
HLA: Human leukocyte antigen
ITIM: Immunoreceptor tyrosine-based inhibitory motif
KIR: Killer cell immunoglobulin-like receptor
LCH: Langerhans cell histiocytosis
MAP: Mitogen-activated protein kinase
MAP2K: Mitogen-activated protein kinase kinase
NK: Natural killer
SHP: Src homology region 2 domain-containing phosphatase.
