# Supplementary material for: Killer cell immunoglobulin-like receptor 2DL4 is expressed in and suppresses the cell growth of Langerhans cell histiocytosis
Source: Oncotarget. 2017 Apr 7;8(23):36964–72. doi: 10.18632/oncotarget.16936 (PMC5514884; doi:10.18632/oncotarget.16936)
Supplement: Supplementary file 1 [file oncotarget-08-36964-s001.pdf]

Killer cell immunoglobulin-like receptor 2DL4 is expressed in and suppresses the cell growth of Langerhans cell histiocytosis

Supplementary Materials

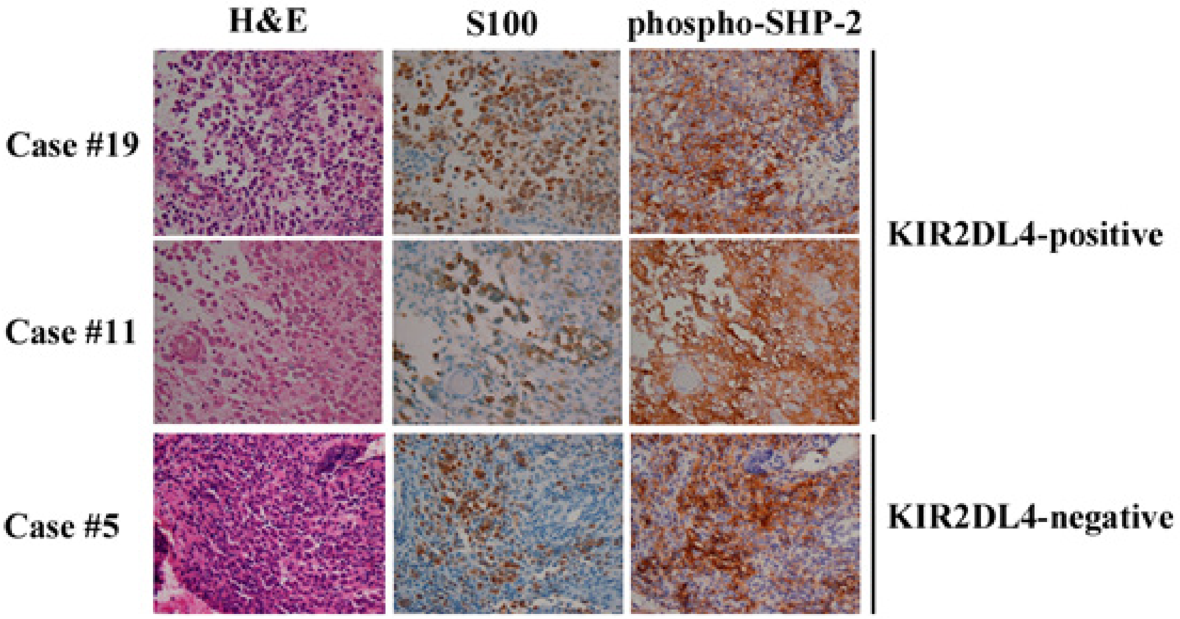

Supplementary Figure 1: Phospho-SHP-2 is expressed in pathological samples of Langerhans cell histiocytosis, independently of KIR2DL4 expression; three representative cases are presented here. Immunohistochemistry (x200).
